# The Role of Genital Tract Microbiome in Fertility: A Systematic Review

**DOI:** 10.3390/ijms23010180

**Published:** 2021-12-24

**Authors:** Salvatore Giovanni Vitale, Federico Ferrari, Michał Ciebiera, Magdalena Zgliczyńska, Agnese Maria Chiara Rapisarda, Giada Maria Vecchio, Alessandra Pino, Giuseppe Angelico, Anna Knafel, Gaetano Riemma, Pasquale De Franciscis, Stefano Cianci

**Affiliations:** 1 Obstetrics and Gynecology Unit, Department of General Surgery and Medical Surgical Specialties, University of Catania, 95123 Catania, Italy; sgvitale@unict.it (S.G.V.); rapisardaagnesemc@gmail.com (A.M.C.R.); 2 Department of Women’s and Reproductive Health, University of Oxford, Headington, Oxford OX3-0BL, UK; f.ferrari.obgyn@gmail.com; 3 Second Department of Obstetrics and Gynecology, Centre of Postgraduate Medical Education, 01-809 Warsaw, Poland; michal.ciebiera@gmail.com (M.C.); zgliczynska.magda@gmail.com (M.Z.); anna.knafel@gmail.com (A.K.); 4 Department of Medical and Surgical Sciences and Advanced Technologies, “ G. F. Ingrassia ” , Anatomic Pathology, University of Catania, 95123 Catania, Italy; giadamariavecchio@gmail.com; 5 Department of Agricultural, Food and Environment, University of Catania, 95123 Catania, Italy; alessandra.pino@unict.it; 6 ProBioEtna, Spinoff of the University of Catania, 95123 Catania, Italy; 7 Unità di Gineco-patologia e Patologia Mammaria, Dipartimento Scienze della Salute della Donna, del Bambino e di Sanità Pubblica, Fondazione Policlinico Universitario A. Gemelli IRCCS, 00168 Roma, Italy; giuangel86@hotmail.it; 8 Obstetrics and Gynecology Unit, Department of Woman, Child and General and Specialized Surgery, University of Campania “Luigi Vanvitelli”, 80128 Naples, Italy; pasquale.defranciscis@unicampania.it; 9 Department of Obstetrics and Gynecology, University of Messina, 98122 Messina, Italy; stefanoc85@hotmail.it

**Keywords:** infertility, microbiome, lactobacillus, IVF

## Abstract

The human microbiome plays a crucial role in determining the health status of every human being, and the microbiome of the genital tract can affect the fertility potential before and during assisted reproductive treatments (ARTs). This review aims to identify and appraise studies investigating the correlation of genital microbiome to infertility. Publications up to February 2021 were identified by searching the electronic databases PubMed/MEDLINE, Scopus and Embase and bibliographies. Only full-text original research articles written in English were considered eligible for analysis, whereas reviews, editorials, opinions or letters, case studies, conference papers, and abstracts were excluded. Twenty-six articles were identified. The oldest studies adopted the exclusive culture-based technique, while in recent years PCR and RNA sequencing based on 16S rRNA were the most used technique. Regardless of the anatomical site under investigation, the *Lactobacillus*-dominated flora seems to play a pivotal role in determining fertility, and in particular *Lactobacillus crispatus* showed a central role. Nonetheless, the presence of pathogens in the genital tract, such as *Chlamydia trachomatis*, *Gardnerella vaginalis*, *Ureaplasma* species, and Gram-negative stains microorganism, affected fertility also in case of asymptomatic bacterial vaginosis (BV). We failed to identify descriptive or comparative studies regarding tubal microbiome. The microbiome of the genital tract plays a pivotal role in fertility, also in case of ARTs. The standardization of the sampling methods and investigations approaches is warranted to stratify the fertility potential and its subsequent treatment. Prospective tubal microbiome studies are warranted.

## 1. Introduction

The human microbiome plays a significant role in determining the health status of every human body and in fact, bacterial communities coexist in mutualistic symbiotic relationships with the host [1,2,3]. The term microbiome was originally defined by Whipps et al. [4] as “a characteristic microbial community occupying a reasonably well-defined habitat which has distinct physio-chemical properties” and today, this definition, is enriched by a dynamic consideration of the microbial activities that result in ecological niches [5]. Variation of the composition of the microbiome can lead to a state of dysbiosis, particularly in case of stress conditions, where the rapid decrease of microbial diversity promotes the expansion of specific bacterial or pathogens [6]. Traditionally, microbiome studies were conducted with culture-based methods that were used to identify bacterial species, but nowadays the introduction of high-throughput DNA sequencing has overcome the limit of the aforementioned approach. In fact, cultured-based methods are still informative, but they only detect a small proportion of organisms that are not representative of the ecological niche under investigation [7]. Similarly, optical magnification techniques have also been used to identify bacteria based on phenotype or morphological details, but today the preferred methods for investigation are sequencing technologies with taxonomy-associated markers genes, such as the 16S rRNA or whole genome sequences [8].

In recent years, the microbiota of various anatomical sites, such as the gastrointestinal (GI) and urogenital tracts, have been investigated, and notably, it was found that the GI tract accounts up to 29% of the whole human microbiome, while the urogenital tract contributes up to 9% [9]. A paradigmatic example is the human vaginal microbiome that is an important site of symbiosis where *lactobacilli* dominate the microbial community and help defend women against infectious disease, hence playing a potential pivotal role in reproductive outcomes, such as fertility and gestational length [9]. Nonetheless, the vaginal microbiome of the mother has an essential well-known role in the initial colonization of the newborn, which impacts their immune system and neurodevelopment [10].

The aim of this systematic review is to report the current evidence regarding the relationship between genital female microbiome and fertility issue and eventual potential implication on assisted reproductive treatments (ARTs).

## 2. Materials and Methods

The literature search for articles regarding microbiota/microbiome/microfilm/microflora and fertility or infertility was performed using three following databases: PubMed/MEDLINE, Scopus, and Embase. The search strategy was properly adapted to each database. Moreover, the authors hand-searched the references of the eligible studies in order to obtain a full view on the topic. Details on the search strategy are summarized in Table 1.

The last search was performed on February 2021 and there were no restrictions on the date of publication. This study aimed to ask the following PICOS questions:

- Population: women or couples with infertility (any type) or non-pregnant condition.
- Intervention: genital tract microbial assessment
- Comparison: optional comparison with microbiome of fertile women.
- Outcomes: composition of the microbial flora correlated with infertility or ARTs failure
- Study design: only full-text original research articles written in English were considered eligible for analysis, whereas reviews, editorials, opinions or letters, case studies, conference papers, and abstracts were excluded.

This review follows the Preferred Reporting Items for Systematic Reviews and Meta-Analyses (PRISMA) guidelines [11]. No Institutional Review Board (IRB) approval was required for this study.

## 3. Results

**Figure 1:**
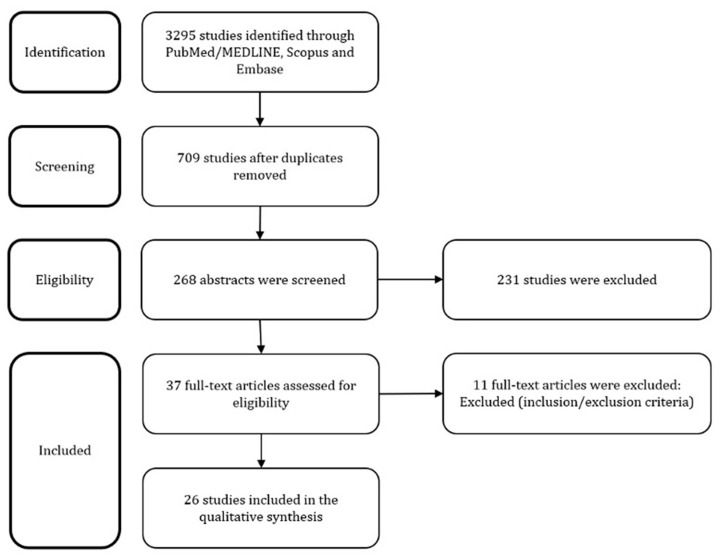
PRISMA Flow Diagram.

The above described search strategy retrieved 3295 articles. After deletion of duplicates and careful overview of the abstracts and full-texts if needed, 20 articles were selected. Moreover, 6 additional articles were found during the hand-searching of its references. Finally, 26 articles that constituted the final pool were discussed in the following paragraphs. The study selection process is reported in Figure 1.

We briefly introduced the sampling and analyses methods and subsequently discussed the findings mainly based on the principal anatomical site under investigation that produces relevant results in every study. A synoptic overview was reported in Table 2.

## 4. Discussions

In this review, we took into account the relevant literature regarding genital tract microbiome and its potential impact on fertility. According to our review, the majority of data suggests a not negligible role of the genital microbiome in infertile women and dysbiosis and the lack of *Lactobacilli* can eventually impair ARTs outcomes. The evaluation of the genital microbiome is not yet standardized nor systematically implemented in any guidelines.

### 4.1. Sampling Methods

The first experiences of microbiome assessment in infertile women were performed with classical methods, such as either vaginal or cervical swabs [13]. The most important steps to be considered are to avoid bacterial contamination, and to sample the exact anatomical site subject of the investigation. Samples can be collected using different sets of swabs, tube, or buffer solution and the sampling can be performed by a trained professional or self-collected. The need of adherence to a strict protocol is of paramount importance to correctly reach the anatomical site without contamination by surroundings ecological niches [9]. The storage of the specimen is also a crucial step that need careful attention until subsequent processing; further, a prompt investigation can decrease the risk of contamination or bacterial overgrowth.

### 4.2. Methods of Analysis

Classical analysis consists of culture-dependant methods; in fact, after a certain period of culturing, different bacterial species can be identified using characteristics cell staining, morphology or observed biochemical reactions [12,14]. Culture-based methods are time-consuming and can provide information only regarding bacteria whose metabolic substrates are provided by the culture terrain. Based on that, it is clear how the main limitation is the lack of a complete and representative scenario of the ecological niche under investigation.

Classification based on gram staining and cell morphology is possible using microscopy of clinical specimens, and today this method is still adopted to identify the clue cell or to assess the Nugent Score in genital microbiome samples. However, this technique is time-consuming and requires not-negligible know-how and training [37].

Over the past decade, there has been an explosion of interest in molecular-based, culture-independent techniques to study the microbiome, and sequencing of 16S ribosomal RNA (rRNA) related genes largely changed the approach to study the microbiome [38]. In fact, 16S rRNA is a component of the 30S small subunit of a prokaryotic ribosome and the genes coding for this component are used to reconstruct phylogenies, due to their slow rates of genomic evolution. The more the sequences of 16S rRNA genes of different bacteria match, the more likely the microbes are related at a higher taxonomic rank; for example, the threshold sequence identity is 94.5% for genera and 86.5% for families [16].

The quantitative polymerase chain reaction (qPCR) is a sensitive method to identify a specific group of bacteria and is a well-established tool for detection, quantification, and typing of different microbial species [39]. qPCR can be used to identify pathogens through the design of an ad-hoc assay that provides fast and high-throughput detection and quantification of target DNA sequences. The main limitation of this technique is the knowledge of gene sequencing of the pathogen under investigation.

After the introduction of next-generation sequencing (NGS) technologies, massive parallelisation of bacterial sequencing of 16S rRNA was possible in combination with bioinformatics pipelines that provided final clustering of the results according to similarity concepts; currently, 99% is proposed as the optimal threshold for similarity to correctly assign a taxonomic unit [29]. The whole genome sequencing (WGS) approach is another interesting opportunity to study not only the sequences of interest, but all the bacterial genomes, the function of the different genes, and to identify novel genes, pathways, structure and organization of genomes [8].

Finally, another method adopted to study microbiomes is the intergenic spaces (IS)-pro technique, which is another amplification approach on the IS regions, whose lengths are specific for each group of bacteria [40]. IS-pro technique is a new qPCR-based profiling technique for high-throughput analysis of the human intestinal microbiota, which combines bacterial species recognition based on the length of the 16S–23S rDNA interspace region with taxonomic classification by labelling PCR primers to phylum-specific fluorescent sequences [41].

### 4.3. Vaginal Findings

The majority of evidence derives from the assessment of vaginal microbiome, that is the easiest anatomical site to sample. The proof of concept of all the studies performed is to identify those patients with an abnormal vaginal microbiome and highlight the differences related to successful ARTs or pregnancy success. The paradigmatic study with culture-based technique is an Indian study, where the vaginal flora of infertile patients was assessed by routine aerobic, anaerobic, and fungal culture with the aim to detect asymptomatic vaginosis and to compare the vaginal microbiota of infertile women versus healthy women. The authors found a prevalence of 28% of asymptomatic bacterial vaginosis (detected by associated pathogens such as *Candida*, *Enterococcus*, and *Escherichia coli*) in infertile patients and, when compared to healthy women, a pronounced lower rate of Lactobacillus colonies [28].

Beside a culture-based approach, the diagnostic performance of qPCR for abnormal microbiome compared to Nugent Score criteria in infertile women was firstly assessed by Haarhr et al. [24] in 130 women. qPCR analysis found an abnormal microbiota in 28% of the population, while Nugent Score detected 21% of patients affected by bacterial vaginosis (BV). Of interest, the sensitivity and specificity of qPCR was both 93% in Nugent-defined BV and only 9% of patients with abnormal microbiota obtained a clinical pregnancy. Similarly, using non culture-based methods, the study of Bernabeu et al. [33] identified different clusters of vaginal microbiome in women with recurrent implantation failure, and precisely a cluster predominated by *Lactobacillus* is associated with the achievement of pregnancy after embryo transfer. On the other hand, a higher rate of *Gardnerella vaginalis* and lower rate of *Lactobacillus* species were correlated with a worse outcome. Again, also the vaginal microbiome profiling with IS-pro technique detected a low load of *Lactobacillus*, and further a high load of *Proteobacteria* or *Lactobacillus jenseni* in those women with failure of pregnancy in a group of 192 women underwent ARTs [32]. Moreover, this technique highlighted two different profiles for *Gardnerella vaginalis*, but only one of these was correlated with low pregnancy rate and overall, only 6% of the women affected by the previous conditions became pregnant after an embryo transfer. Curiously, the high abundance of *Lactobacillus crispatus* (greater than 60%) was correlated with a worse outcome of ARTs and hence generalization regarding the possible beneficial effects of *Lactobacilli* on fertility should be avoided, given that different species of the latter may differently affect ART outcomes. Campisciano et al. [21] adopted the NGS approach to compare the vaginal microbiome of idiopathic and non-idiopathic infertile women, healthy women, and women affected by BV with the aim to detect suitable biomarkers for infertility. The vaginal microbiome of idiopathic infertile women was similar to BV-associated microbiome. Furthermore, *Lactobacillus iners* resulted as a marker of healthy vaginal microbiome, while *Lactobacillus crispatus* levels were lower in idiopathic infertile women when compared to healthy women and these findings is only partially in contrast to the previous study. In fact, on one hand, the drop of *Lactobacillus crispatus* may lead to an increased susceptibility to pathogens, hence favouring BV; on the other hand, the simple presence of the latter is not a favourable factor, but the degree of domination of *Lactobacillus crispatus* in the vaginal microbiome is probably the key of lecture.

An interesting study by Borovkova et al. [18] clarified the influence of the sexual intercourse on the genital tract microbiome of infertile couples. The study protocol was based on PCR analysis of self-collected vaginal samples taken 3–5 days before and 8–12 h after intercourse and semen samples collected during the subsequent menstrual period of the partner. *Ureaplasma parvum* was found in 59% of women and its prevalence was higher in women whose partners had inflammatory prostatitis; nonetheless, the same pathogen was found in half of the male partners. After intercourse, a median of four and two new species emerged, respectively, and disappeared in women, and the authors noted that these changes were less frequent in the presence of a normal vaginal microbiota and more prominent in the partners of men affected by inflammatory prostatitis, arguing that this microbial shifting can interfere with fertilization.

The embryo implantation rate was not significantly decreased by the presence of BV in a study of 307 women who underwent in vitro fertilization (IVF), whose vaginal microbiome was investigated with a validated diagnostic test by qPCR using sterile cytobrush and cotton swabs [19]. In this study, the overall prevalence of BV was 9%, with a greater prevalence (22%) in those who performed vaginal douching. Not only was the rate of embryo implantation similar, but the clinical pregnancy rate was also not statistically different, and no differences were noted in terms of worse obstetric outcomes, so the authors can only conclude that the only advise for women is to not perform vaginal douching.

### 4.4. Cervical Findings

One of the first studies [13] describing the normal flora of the cervix of different populations of women, respectively infertile, pregnant, and in labour patients found no difference in the distribution of the bacterial species; however, in infertile women, the authors reported the lowest proportion of both aerobic and anaerobic bacteria and further, exclusive anaerobic flora was found in more than half of the group of infertile women, indicating that the human cervix flora should be regarded as a complex of interacting and competing bacteria. Previously, Hok et al. [12] reported similar findings, affirming that *Mycobacterium tuberculosis* was the only one with a higher occurrence in infertile patients; again, bacterial culturing [14] on cervical swabs, performed for cervicitis, failed to show different cervical flora in infertile women, even though 16% of these women were found positive for *Chlamydia trachomatis* antibodies on the serum. A further confirmation derived from the study of Cheong et al. [36] that assessed the prevalence of the aforementioned pathogen using rRNA metagenomic sequencing on endocervical swabs and found that the large majority of infertile women was infected with *Chlamydia trachomatis*, in contrast with a lower prevalence of the infection in the fertile group of women (88% vs. 28%) after correction of the results according to demographics parameters. Moreover, many further associated pathogens were isolated more frequently in those women with *Chlamydia* trachomatis infection. Given the evidence that *Chlamydia trachomatis* is linked with infertility, Graspeuntetner et al. [27] adopted 16S rRNA sequencing on cervical specimen and parallelly investigated the presence of anti-*Chlamydia* antibodies in patients with infectious infertility, non-infectious infertility and healthy volunteers. A higher rate of *Chlamydia trachomatis* was observed in infectious infertility women when compared to non-infectious group, while a progressive decrement of *Lactobacillus* species was detected in healthy volunteers, non-infectious, and infectious infertility women (78.3%, 69%, and 58%), respectively; on the contrary, *Gardnerella vaginalis* was seen with an increasing trend in the same groups (5%, 6%, and 10%) and a similar trend was observed also for *Prevotella* and *Sneathia*.

Bacteria culturing of cervical canal swab performed before embryo transfer in 204 women demonstrated a significant correlation between the presence of any gram negative colonization and the failure of conception; notably, women with sterile cervical culture or demonstrating *Lactobacillus* had a higher chance of getting pregnant [17]. Similarly, a French study [16] instead assessed the bacteriology state of the catheter tip used as an endocervical probe before embryo transfer in 279 infertile women underwent IVF and correlated the results with successful clinical pregnancies. In the women with positive culture, the authors registered a predominance of *Escherichia coli* and *streptococcus* species with clinical and ongoing pregnancy rates and implantation rates significantly lower when compared to women with negative culture (24% versus 37%; 17% versus 28%; and 9% versus 16%, respectively).

### 4.5. Endometrial Findings

The first study assessing the microbiome of the endometrium in infertile women was conducted by Ilesanmi et al. [15] with a culture-dependent analysis based on endometrial samples collected with sterile precautions. None of the cultures of endometrial biopsy specimens yielded any growth and the Gram stains were all negative, both for organisms and pus cells and hence authors concluded that this practice is not useful for this clinical scenario. Conversely, an explorative study [22] on 19 non pregnant-women, including those affected by sub-fertility condition, adopted PCR analysis and RNA sequencing to investigate the putative presence of uterine microbiome and found that 90% of the patients had a predominant presence of *Bacteroides* and *Pelomonas* taxa, and in a few cases, they noted the presence of *Lactobacillus crispatus* and *Iners* associated more frequently with *Bacterioides* species. Moreno et al. [23] demonstrated in women undergoing IVF the existence of an endometrial microbiota that is highly stable during the acquisition of endometrial receptivity, but differently associated with adverse reproductive outcomes based on its composition. In their study, the authors compared the results of 16S rRNA analysis of paired samples of endometrial fluid and vaginal aspirates in pre-receptive and receptive phases within the same menstrual cycle and correlated them with the reproductivity outcomes. Regardless of hormonal status, the comparison with the vaginal microbiota demonstrated that the endometrial microbiota is not a carryover from the vagina, because some bacterial genera present in the endometrium were not in the vagina of the same subject, and vice versa. This approach permitted identification of the existence of vaginal and endometrial bacterial communities that are not identical in every woman. The endometrial microbiota with a non-*Lactobacillus*-dominated flora (<90% of *Lactobacillus* species with >10% of other bacteria) was associated with decreased implantation, pregnancy, ongoing pregnancy, and live birth rates. The authors suggested that the lack of *Lactobacillus*-dominated flora can be considered as an emerging cause of implantation failure and pregnancy loss. A subsequent pilot study conducted in 102 infertile Japanese women [30] assessed and compared the endometrial versus vaginal microbiota by 16S rRNA sequencing. The authors included healthy volunteers as the control group, which allowed them to demonstrate a high stable rate of *Lactobacilli* both in endometrial and vaginal samples and also in the following cycle, confirming that *Lactobacillus*-dominated flora is independent of hormonal changes during the fertile period. The authors investigated the endometrial versus vaginal microbiome of different groups of patients, finding rates of *Lactobacillus*-dominated flora of 38% (30/79) versus 44.3% (44/79), respectively, in IVF patients, and 73.9% (17/23) vs. 73.9% (17/23) in the non-IVF patients. Considering the percentage of abnormalities in the microbiome of IVF patients, compared to the non-IVF patients or healthy volunteers, the authors believed that endometrial microbiome might be related to unexplained recurrent implant failures. Another comparative study [35], characterized by qPCR analysis the endometrial fluid and vaginal microbiome in infertile women affected by recurrent implant failures (RIF) versus those undergoing their first IVF attempt. No differences among endometrial and vaginal microbiome in the detection of specific bacterial species within the same individual were detected; however, endometrial microbiome presented a higher degree of microbial diversity when compared to its vaginal counterpart. Surprisingly, *Lactobacillus*-dominated flora was seen more frequently in the RIF group, than in the control group, although without significant statistical difference, both in the endometrial fluid and vaginal samples. *Gardnerella Vaginalis* was detected more frequently in endometrial fluid of the RIF group, even though without statistical significance; noteworthy, *Burkholderia* was detected in 25% of the RIF group and none in the control group. A great limitation of this study is the unpredictable longitudinal RIF in the control group, making the results less generalizable. A study by Liu et al. [34] systematically compared the endometrial microbiota in 130 infertile women with and without chronic endometritis (CE), diagnosed by sequencing of 16S rRNA. CE was associated with a statistically significant higher abundance of 18 bacterial taxa in the endometrial cavity; relative abundance of *Lactobacillus* was greater in non-CE patients (1.89% vs. 80.7%) with a less abundance of *Lactobacillus crispatus* in the CE group of patients while *Anaerococcus* and *Gardnerella* were negatively correlated with the relative abundance of Lactobacillus in CE microbiome.

### 4.6. Miscellanea Findings

An Indian study by Mishra et al. [26] included 288 infertile couples, performing either cervical or high vaginal swab and semen samples for culture analyses. A high prevalence of *Escherichia coli* was implicated in primary infertility, even though it was not the only isolated pathogen; hence, the authors suggested adopting therapeutic managements strategies of couples with primary infertility such as antibiotics and a parallel treatment to avoid cross-retransmission. A pilot study by Wee et al. [31] compared the microbiome of the vagina, the cervix, and endometrium of infertile women in comparison to those of women with a history of fertility, using 16S rRNA amplification, moreover, they conducted an ancillary investigation into the endometrial expression of selected genes using PCR analysis. *Lactobacillus* genus was the most frequently observed overall and the abundance of taxonomic units was the same both in cervical and vaginal specimens; the dominant microbiome was consistent also in regards to the endometrium, even though in different relative proportions. There was a trend for infertile women that demonstrated *Ureaplasma* in the vagina and *Gardnerella* in the cervix. The test for the expression of selected genes in the endometrium failed to show any correlations with case–control status, or with microbial community composition, although Tenascin-C expression was correlated with a history of miscarriage.

Pelzer et al. [20] compared the microbiome of human follicular fluid of 202 infertile women with vaginal sample counterparts using culturing and PCR analysis. They found that the presence of *Lactobacillus* species in human follicular fluid was associated with embryo maturation and transfer, and conversely the presence of contaminants (bacteria detected also in the matched vaginal sample) affected adversely the IVF outcomes without however identifying a single species associated with decreased fertilization rates.

Notably, a couple of studies included in our review undertook both RNA sequencing on the distal part of the catheter used for embryo transfer [25] and one of them also added a PCR analysis [29]. In their analysis, the studies failed to detect a difference in the prevalence of *Lactobacilli* and lactic acid-producing bacteria among women with ongoing pregnancy against who did not have a pregnancy. These couple of studies represent a proof of concept for the characterization of the microbiome at the time of embryo transfer and could potentially be prospectively employed to understand the need of probiotics in a subsequent successful pregnancy event.

## 5. Conclusions

The available data suggest a not negligible role of the genital microbiome in infertile women and further, dysbiosis and the lack of *Lactobacilli* can potentially impair ARTs and partially explain implantation failures. A complex interaction of the *Lactobacillus* species plays a pivotal role for the equilibrium of the normal vaginal flora and in particular appropriate levels of *Lactobacillus crispatus* are involved as protective factor for asymptomatic BV (mostly sustained by *Ureaplasma* and *Gardnerella vaginalis*) that is recognized as a potential impairing factor of fertility. Similarly, the presence in the cervical flora of Gram-negative bacteria, *Chlamydia trachomatis* and *Gardnerella vaginalis*, and the eventual coexistent lack of *Lactobacillus* is linked with infertility condition. The lack of a *Lactobacillus*-dominated flora in the endometrium seems linked with recurrent implant failures, and the latter is supported by the prevalence of *Bacteroides* species in the endometrium of non-pregnant women, given the fact that hormonal changes do not impact the variety of endometrial microbiome, including the relative abundance of *Lactobacilli*. Of note, *Gardnerella vaginalis* was also isolated in the endometrium of infertile women. Embryo transfer seems not influenced by vaginal dysbiosis, even though the strength of this affirmation is very weak, whereas the success of the embryo transfer is conversely impacted by abnormal cervical flora.

Another point of discussion is the total lack of data regarding microbiome of the tube(s); in fact, we failed to identify studies investigating this anatomical site. Of course, we believe that the sampling of the tube is a more invasive procedure that cannot be performed as easily as a vaginal swab, even though an outpatient procedure with an in-office hysteroscope might be feasible and well tolerated by the woman. In literature, we found examples of brush cytology of the fallopian tube [42,43,44] using minimally invasive hysteroscopes [45,46,47,48,49], even though in the setting of prevention of ovarian cancer [43,45]. A previously reported study [20] investigated the contamination of follicular fluid by vaginal pathogens, hence, providing the rational for tubal microbiome sampling in infertile women. Similarly, comparative microbiome studies of the different anatomical sites of the genital tract, namely vagina, cervix, endometrium, tube(s), and follicular fluid with 16S rRNA analyses can represent an exciting unexplored field of research [46,50].

Microbiome profiling requires international consensus regarding sampling and investigation analyses that should be performed without culture-dependent methods, and large prospective studies with homogeneous inclusion and exclusion criteria are needed to explore this field with a robust clinical and translational outlook.

## Figures and Tables

**Table 1:** Search strategy for used databases.

Database	Number of Retrieved Studies	Search Strategy
PubMed/MEDLINE	650	(“Microbiota” [Mesh] OR microbiot * OR microbiom * OR microfilm * OR flora OR microflor *) AND (“Infertility” [Mesh] OR infertility OR fertility OR sterility)
Embase	963	(microbiot * OR microbiom * OR microfilm * OR ‘flora’/exp OR flora OR microflor *) AND (infertility OR fertility OR sterility)
Scopus	1682	TITLE-ABS-KEY ((microbiot * OR microbiom * OR microfilm * OR flora OR microflor *) AND (infertility OR fertility OR sterility))

**Table 2:** Synoptic overview of the articles included in the review.

Authors	Year	Country	Aim	Basic Data on Studied Infertile Women/Couples	Specimen Type	Diagnostic Method	Data on Cultures Growth	Impact on Fertility or Infertility Treatment Outcomes	Additional Information
Hok et al. [12]	1967	Indonesia	To examine the incidence of various bacteria in endocervical mucus obtained from the internal os of women with gynecological issues.	112 infertile woman qualified for tubal insufflation	Endocervical mucus	Bacteria culturing	No differences between groups except higher occurrence of Mycobacterium tuberculosis in infertile patients; the growth pattern in infertile patients was as *Streptococcus*—29%; *Gaffkya tetragena*—20%; *Bacillus*—15%; *Pseudomonas aeruginosa*—15%; *Proteus*—13%; *Alcaligenes faecalis*—6%; *Diphtheroid* rods—5%:	In 3% cases of infertility, *Mycobacterium tuberculosis* was found and was expected to be the reason of infertility.	
Moberg et al. [13]	1978	Sweden	To describe the predominant cervical bacterial flora in infertile, early-pregnancy and labour patients	47 infertile women	Cervical swabs	Bacteria culturing	Alpha-hemolytic streptococci—23% *Streptococci* group B—0% *Streptococci* group C—5% *Streptococci* group D—18% *Staphylococcus aureus*—4% *Staphylococcus epidermidis*—41% *Enterobacteriaceae*—9%	The largest proportion of patients with both aerobic and anaerobic bacteria was found in the labour group, followed by the early pregnancy and the infertile groups. The same relation between the groups was noted for aerobic bacteria only; only anaerobic bacteria was found in 51% of infertile patients, 26% of early pregnancy and 0% in labour group	-
Koskimies et al. [14]	1981	Finland	To examine the cervical factor using microbiologic, cytologic, colposcopic, and histologic investigations in a group of women with reproductive failure and clinically “inflame cervixes”	52 infertile women	Cervical swabs and serum	Bacteria and yeast culturing and anti-chlamydial antibodies immunofluorescence	*Chlamydia trachomatis*—8% *Escherichia coli*—10% *Staphylococcus epidermidis*—21% *Lactobacillus*—8% *Enterococcus*—6% *Staphylococcus aureus*—4% *Bacteroidea* sp.—4% *Bacteroides fragilis*—2% Group B *streptococcus*—2% *Peptostreptococcus*—2% “Normal mixed flora”—13% No growth—21% *Candida albicans*—23% *Trichomonas vaginalis*—2% *Neisseria gonorrhoeae*—0	Women examined for infertility have been shown to have significantly higher levels of anti-chlamydial antibodies than the controls	No testing for *Ureaplasma urealyticum* was performed
Taylor & Ilesanmi et al. [15]	1995	Nigeria	To examine the microbial flora, if any, of the endometrium of infertile women and re-appraise the value of endometrial and vaginal bacterial cultures in the assessment of the infertile woman	73 infertile women 12 (16.4%) primary 61 (83.6%) secondary	Endometrial biopsy	Histological examination and bacteria culturing	None of the cultures yielded any growth and the Gram stains were all negative, both for organisms and pus cells.	-	No testing for *Chlamydia* was performed.
Fanchini et al. [16]	1998	France	To analyze bacteriologically the catheters used for determining cervical obstruction before ET for the presence of microorganisms and to investigate the possible consequences on the outcome of IVF-ET	279 infertile women undergoing controlled ovarian hyperstimulation and IVF-ET	Samples from ET catherer	Bacteria culturing	51% cultures were positive—Mostly *Escherichia coli* (64%) and *Streptococcus* species (8%) Only aerobic—86% Only anaerobic—5% Both aerobic and anaerobic—9% Gram-negative—62% Gram-positive—25% Both gram-negative and gram-positive—13%	Clinical, ongoing pregnancy and implantation rates were lower in the positive than in the negative culture group (24% vs. 37%; 17% vs. 28%; and 9% vs. 16%, respectively)	No testing for *Chlamydia* or *Mycoplasma hominis* was performed
Salim et al. [17]	2002	Israel	To examine whether the nature of bacterial flora, found in the uterine cervical canal at embryo transfer, is associated with the rate of conception in assisted reproductive techniques	204 patients who underwent embryo transfer 68% were of fresh embryos, following recent vaginal oocyte retrieval and prophylactic antibiotic therapy, and 32% of frozen–thawed embryos	Cervical canal swab	Bacteria culturing	In 75 patients (37%) sterile cervical cultures or lactobacillus were recorded; No difference in colonization was found between women who underwent frozen–thawed versus fresh embryo transfer (57 and 67% respectively); *Escherichia coli* was found in 8.5% of the positive cultures	Any Gram-negative colonization was associated with no conception; From 75 sterile culture patients 31% conceived; among the 129 in whom any pathogenic micro-organism was recovered only 16% conceived	
Borovkova et al. [18]	2011	Estonia	To clarify the influence of sexual intercourse on partner’s genital tract microbiota in infertile couples	17 infertile couples	Self-collected vaginal samples taken 3–5 days later before intercourse and 8–12 h after intercourse and semen samples collected during menstruation of the partner	PCR analysis	*Lactobacilli*—88% Coagulase (−) *staphylococci*—88% *Ureaplasma parvum*—59%; its prevalence was higher in these women whose partner had IP (80% vs. 50%) *Corynebacteria*—76% Anaerobic Gram (+) bacteria—76% *Streptococci*—65% *Trichomonas vaginalis*—6%	-	After the intercourse, median 4 new species emerge in one woman and median 2 species disappear; this tendency was more prominent in the partners of IP patients
Mangot-Bertrand et al. [19]	2012	France	To assess BV prevalence for infertile patients treated by IVF and its impact on the pregnancy rate	307 women undergoing IVF	Vaginal sampling performed with sterile cytobrush and two sterile cotton swabs	Nugent score and RT-PCR	The prevalence of BV in the whole study group was 9%; among women who performed vaginal douching—22%; whereas among patients who did not douche—8%	The embryo implantation rate does not decrease significantly between the two groups (36% in group 1 BV− vs. 28% in group 2 BV+) nor does the clinical pregnancies rate (33% vs. 28% (both *p* > 0.05)	Regarding ongoing clinical pregnancies, authors did not observe any significant difference between the two groups concerning early miscarriage rate, PROM, preterm labour, gestational age at delivery, mode of delivery or birthweight
Pelzer et al. [20]	2013	United States	To test human follicular fluid for the presence of microorganisms and to correlate these findings with the IVF outcomes	263 couples 202 infertile women during IVF procedures	Follicular fluid samples and vaginal swabs	Culturing and PCR analysis	Microorganisms were detected within 100% of cultured vaginal swabs; *Lactobacillus* spp., *Bifidobacterium* spp. And *Staphylococcus* spp. were the most prevalent species detected in the lower genital tract specimens of all women. The culture analyses revealed that cultivable bacterial species (1–5 species) were present in 99% of follicular fluids tested. The rates of colonization in infertile women ranged from 24% to 37% with no evidence of a difference in colonization rates across cohorts based on the causes of infertility	Adverse IVF outcomes were associated with microbial colonization of follicular fluid. No single species was associated with decreased fertilization rates; however, the presence of *Lactobacillus* spp. in both right and left follicles was associated with higher rates of embryo transfer; the presence of *Propionibacterium* spp. and *Streptococcus* spp. in right follicles was associated with poor embryo transfer rates	Microorganisms isolated from follicular fluids were classified as: (1) ‘colonizers’ if microorganisms were detected within the follicular fluid, but not in vaginal swab; or (2) ‘contaminants’ if microorganisms detected in the vagina were also detected within the follicular fluid; The authors found significant differences in the microbiome of both (left and right) ovaries
Campisciano et al. [21]	2016	Italy	To compare the vaginal microbiome of idiopathic and non-idiopathic infertile as well as fertile women to identify bacterial species suitable as biomarkers	27 infertile women attending the ART	Cervical–vaginal fluids	V3-16S rDNA sequencing	*Lactobacillus iners*, *Lactobacillus crispatus*, and *Lactobacillus gasseri* distinguished idiopathic infertile women from the other groups. In these group, a microbial profile similar to that observed in BVwomen has been detected; in Idiopathic we observe a lack of *Fusobacteria*, which are present only in Vaginosis, and the presence of *Clostridia*, missing only in Control. Infertile has not *Bacteroidia* and *Tenericutes*, which are present in the other three groups, mainly in Idiopathic and Vaginosis. At the lowest taxonomic level, among species belonging to *Lactobacilli* (*Firmicutes*), *Lactobacillus gasseri*, *Lactobacillus iners*, and *Lactobacillus crispatus* are the most abundant in Idiopathic. *Lactobacillus gasseri* is overrepresented in Idiopathic compared to the other three groups; *Lactobacillus iners* is underrepresented in Idiopathic compared to Control, and its abundance decreases in Vaginosis and Infertile. *Lactobacillus crispatus* is more abundant in Control than Idiopathic, while Vaginosis shows the lowest abundance. *Veillonella* (*Firmicutes*) and *Staphylococcus pasteuri/warneri* (*Firmicutes*) are common to Idiopathic and Vaginosis. Among *Actinobacteria*, *Gardnerella vaginalis* is underrepresented only in Control. *Atopobium vaginae* (*Actinobacteria*) and *Prevotellabivia* (*Bacteroidetes*) are shared between Idiopathic and Vaginosis.		
Verstraelen et al. [22]	2016	Belgium	To investigate the putative presence of a uterine microbiome in a selected series of non-pregnant women through deep sequencing of the V1-2 hypervariable region of the 16S ribosomal RNA (rRNA) gene	19 patients (various reproductive conditions, including subfertility)	Endometrial sample	PCR analysis RNA sequencing	In 90% of subjects, the community was fairly similar, with different species of *Bacteroides* and *Pelomonas* taxa. In six cases, additional high abundance of *Lactobacillus crispatus* and *Lactobacillus iners* was found in the presence of *Bacteroides*; 15 phylotypes were present in all samples; in 90% of the women community architecture was similar in as much *Bacteroides xylanisolvens*, *Bacteroides thetaiotaomicron*, *Bacteroides fragilis* and an undetermined *Pelomonas* taxon constituted over one third of the endometrial bacterial community		
Moreno et al. [23]	2016	Spain	To test the existence of an endometrial microbiota that differs from that in the vagina and analyze the impact of the endometrial microbial community on reproductive outcome in infertile patients undergoing IVF	35 infertile patients	Endometrial fluid	RNA sequencing	The most represented genus was *Lactobacillus* (72% of identified bacteria); *Gardnerella* (12.6%), *Bifidobacterium* (3.7%), *Streptococcus* (3.2%), and *Prevotella* (0.866%) were the other most common genera	The presence of a non-*Lactobacillus*-dominated microbiota in a receptive endometrium was associated with significant decreases in implantation (61% vs. 23%) pregnancy (71% vs. 33%) ongoing pregnancy (59% vs. 13%) and live birth (59% vs. 7%) rates	If bacterial communities from paired endometrial fluid and vaginal aspirate samples within the same subjects were interrogated, different bacterial communities were detected between the uterine cavity and the vagina of subjects
Haahret al. [24]	2016	Denmark	To characterize what is the diagnostic performance of qPCR assays compared with Nugent scoring for abnormal vaginal microbiota and for predicting the success rate of IVF treatment	130 infertile women	Vaginal swabs	Nugent’s criteria and PCR analysis	The prevalence of BV defined by Nugent score was 21%,whereas the prevalence of an abnormal vaginal microbiota was 28% defined by qPCR; there were high concentrations of *Gardnerella vaginalis* and/or *Atopobium vaginae*	Abnormal vaginal microbiota may negatively affect the clinical pregnancy rate in IVF patients; only 9% with qPCR defined abnormal vaginal microbiota obtained a clinical pregnancy	The qPCR diagnostic approach had a sensitivity and specificity of respectively 93% and 93% for Nugent-defined BV
Franasiaket al. [25]	2016	United States	To characterize the microbiome at the time of embryo transfer	33 infertile women 18 ongoing pregnancies 15 not	Distal part of transfer catheter used for embryo transfer	16S ribosomal subunit hypervariable region analysis with next-generation sequencing	There were a total of 278 different genus calls present across patient samples; Flavobacterium and Lactobacillus represent the majority of the bacterium seen in both groups	Lactobacillus was the most often found species for both pregnant and not; there were no differences in microbiomes between ongoing and non-ongoing pregnancy	
Mishra et al. [26]	2017	India	To know the prevalence of microorganisms in the infertile couples of a tertiary caring teaching hospital	288 infertile couples 68% primary 32% secondary Male factor—27%, Female factor—50% Both—5% Unexplained—18%	The endocervical swabs or high vagina swabs	Bacteria and yeast culturing	65% of women from infertile with cultures growth *Escherichia coli*—27% *Staphylococcus aureus*—23% *Pseudomonas* sp. 21% Coagulase (−) *Staphylococci*—17%, *Candida albicans*—9% *Klebsiella* sp.—3%	-	-
Graspeuntner et al. [27]	2017	Germany	To characterize the microbial pattern in females diagnosed with ININF in comparison to females with nININF, FSW and healthy controls	47 infertile women 26 females women with nININF 21 women with ININF	Three swabs from the cervix and serum samples	16S amplicon sequencing and anti-chlamydial antibodies immunobloting	Females with ININF had higher frequency of previous *Chlamydia trachomatis* infections in comparison to fertile and nININF; while on average 78.34% of all sequence reads in fertile females belong to *Lactobacillus*, the percentage is reduced to 69% in nININF, 58% in ININF; the relative read count of the genus *Gardnerella* increased from 5% in fertile females, to 6% in nININF and up to 10% in ININF and the same trend is observed for the genera *Prevotella* and *Sneathia*	Authors established a model to predict the underlying cause of infertility by using the following parameters: (1) detection of pathogens by PCR and cultivation, (2) serological status of *Chlamydia trachomatis* IgG/IgA and (3) the first ten taxa found in microbiota sequencing; it correctly predicted 17 of 18 ININF—Area under curve: 0.978	-
Babu et al. [28]	2017	India	To compare the vaginal flora and analyse the incidence of asymptomatic vaginosis among healthy women and in women with infertility problems	116 infertile women	Vaginal swabs	Bacteria culturing	The most dominant flora was *Candida* spp. (27%), *Enterococcus* (23%) followed by Gram negative bacilli such as *Escherichia coli* (14%). The percentage of *Lactobacillus* was relatively low (4%). Asymptomatic vaginosis was present in 28%		
Tao et al. [29]	2017	United States	To establish the validation of taxonomic identification and characterize endometrium microbiome by analyzing embryo transfer catheter tips	70 infertile women	Distal part of transfer catheter used for embryo transfer	PCR analysis RNA sequencing	*Lactobacillus* spp. were detected in all of the samples; Among 70 samples, 33 samples contained over 90% of *Lactobacillus* abundance and 50 samples contained over 70% of *Lactobacillus* abundance, which was consistent with the dominance of *Lactobacillus* in the lower reproductive tracts; *Corynebacterium* spp. were detected in 40 out of 70 patients. *Bifidobacterium* spp. were detected in 15 out of 70 patients and *Staphylococcus* spp. were detected in 38 out of 70 patients. Other lactic acid-producing bacteria from the genera *Streptococcus* were present in 38 out of 70 patients	-	The metagenomics workflow provides a rapid and sensitive method to identify bacteria in clinical embryo transfer specimens
Kyono et al. [30]	2018	Japan	To analyze the endometrial and vaginal microbiome among a Japanese infertile population and its impact on implantation	102 infertile patients 79 IVF 23 non-IVF	Endometrial fluid and vaginal discharge samples collected using an intrauterine insemination catheter	PCR and pyrosequencing of V4 of the bacterial 16S rRNA genes	The *Lactobacillus*-dominated microbiota (>90% *Lactobacillus* spp.) in the endometrium vs vagina was 38% vs. 44% in the IVF patients, 74% vs. 74% in the non-IVF patients, and 86% vs. 86% in the healthy volunteers. The major taxonomies were *Gardnerella*, *Streptococcus*, *Atopobium*, *Bifidobacterium*, *Sneathia*, *Prevotella*, and *Staphylococcus*; the median percentage of the endometrial *Lactobacilli* in the IVF patients was lower than that of the non-IVF patients and healthy volunteers (64% vs. 96% vs. 99.5%); the percentage of LD endometrial status was also the lowest in the IVF group (38% vs 74% vs 86%)	A considerable percentage of non-*Lactobacillus*- dominated (NLD) microbiota was found in the endometrium of Japanese infertile women. During the study, 18 patients achieved pregnancy: 3 natural conceptions and 15 by single vitrified-warmed blastocyst transfer. The median percentages of the endometrial and vaginal *Lactobacilli* in those pregnant cases were 96% and 98%. There were seven NLD cases (six IVF and one non-IVF) who achieved pregnancy—5 ongoing, 1 early miscarriage and 1 lost to follow-up	
Wee et al. [31]	2018	Australia	To examine the vaginal, cervical, and endometrial microbiota for women with a history of infertility compared to women with a history of fertility	15 infertile patients	Vaginal, cervical, and endometrial samples	PCR analysis RNA sequencing	The genus *Lactobacillus* was most frequently observed. The most abundant taxonomic units were the same in both cervical and vaginal specimens for all 24 of the 33 women where both were sequenced	Infertile women more often had *Ureaplasma* in the vagina and *Gardnerella* in the cervix	Tenascin-C expression correlated with a history of miscarriage
Koedooder et al. [32]	2019	Netherlands	To determine if the presence or absence of certain vaginal bacteria associated with failure or success to become pregnant after an in IVF or IVF-ICSI) treatments.	192 women undergoing IVF or IVF-ICSI treatment	Self-collected vaginal swab	Microbiome profiling with the use of interspace profiling (IS-pro)	A relatively low load of *Lactobacillus*, a high load of *Proteobacteria* or *Lactobacillus jensenii* was correlated with failure to become pregnant. We found that *Gardnerella vaginalis* strains were characterized by two distinct ISprofiles. Only one of these IS-types was correlated with low pregnancy rate, namely IS-pro type 1 (IST1). Combined observations resulted in a predictive algorithm for failure to become pregnant with the following parameters: relative *Lactobacillus* load <20%, relative load of *Lactobacillus jensenii* >35%, presence of *Gardnerella vaginalis* IST1 or *Proteobacteria* >28% of total bacterial load and such situation was called unfavourable microbiome profile—6% of these women became pregnant after a fresh ET In the group with *Lactobacillus crispatus* abundance of ≥60%, 24% of women became pregnant. In the group with *Lactobacillus crispatus* abundance <60%, 53% (50 of 95) of women became pregnant. All women who did have a favourable profile (158/192 women, 82%) could be stratified into groups with a high and an average chance of pregnancy based on relative abundance of *Lactobacillus crispatus* alone. The prediction model identified a subgroup of women (18%) who had a low chance to become pregnant following fresh ET. This failure was correctly predicted in 32 out of 34 women based on the vaginal microbiota composition, resulting in a accuracy of 94% (sensitivity, 26%; specificity, 97%). Additionally, the degree of dominance of *Lactobacillus crispatus* was an important factor in predicting pregnancy. Women who had a favourable profile as well as <60% *Lactobacillus crispatus* had a high chance of pregnancy: more than half became pregnant.		
Bernabeu et al. [33]	2019	Spain	To investigate if the vaginal microbiome influences the IVF outcome	31 patients undergoing ART	Vaginal samples	V3 V4 region of 16S rRNA analysis	*Lactobacillus* spp. standing out as the most prevalent genus. It was majorly represented by *Lactobacillus crispatus* (47%), *Lactobacillus helveticus* (23%), *Lactobacillus iners* (22%), and *Lactobacillus jenseii* (4%); The cluster analysis identified two main clusters: the first encompassed the genera *Lactobacillus*, *Gardnerella*, *Clostridium*, *Staphylococcus*, and *Dialister*, and the second included all other genera.	Samples from women who achieved pregnancy showed a greater presence of *Lactobacillus* spp.; In women who achieved pregnancy, the microorganisms mainly from the first cluster were detected.	
Liu et al. [34]	2019	China	To systematically compare the endometrial microbiota in infertile women with and without chronic endometritis, as diagnosed by a quantitative and reference range-based method	130 infertile women	Endometrial biopsy and fluid	RNA sequencing	The median relative abundance of *Lactobacillus* was 2% and 81% in the chronic endometritis and non-chronic endometritis microbiotas, respectively. *Lactobacillus crispatus* was less abundant in the chronic endometritis microbiota. Eighteen non-*Lactobacillus* taxa including *Dialister*, *Bifidobacterium*, *Prevotella*, *Gardnerella*, and *Anaerococcus* were more abundant in the chronic endometritis microbiota and of these, *Anaerococcus* and *Gardnerella* were negatively correlated in relative abundance with *Lactobacillus*		Chronic endometritis was associated with a higher abundance of 18 bacterial taxa in the endometrial cavity
Kitaya et al. [35]	2019	Japan	To characterize the microbiota in the endometrial fluid and vaginal secretions in women with RIF	46 infertile patients 28 with with a history of RIF 18 infertile patients undergoing the first IVF attempt	Vaginal swab and endometrial fluid	PCR analysis	There were no significant differences in the detection rate of the specific bacterial species in the VS microbiota between the two groups. *Lactobacillus* dominated endometrial fluid microbiota, defined by >90%; *Lactobacillus* genus status, was observed at a higher rate in the RIF group (64%) than in the control group (39%) without statistical significance; In vagina 67.9% women in the RIF group and 44.4% in the control group represented *Lactobacillus*-dominated microbiota. The detection rate of *Gardnerella* in the EF microbiota was 39.3% in the RIF group and 27.7% in the control group. *Burkholderia* was not detected in any of the EF microbiota in the control group but was detectable in 25% of the RIF group. *Burkholderia* was not detectable in infertile women undergoing the first IVF-ET attempt but in a quarter of those with a history of RIF		
Cheong et al. [36]	2019	Malaysia	To evaluate the alteration of endocervical microbiome in association with *Chlamydia trachomatis* infection among a cohort of women in Malaysia	34 infertile women	Endocervical swabs	16S rRNA metagenomic sequencing	A total of 40 out of 70 participants were infected with genital *Chlamydia trachomatis* based on the diagnostic test result; higher level of *Delftia*, *Streptococcus*, *Pseudomonas*, *Cloacibacterium*, *Prevotella*, *Veillonella*, *Megasphaera*, *Ureaplasma*, and *Ralstonia* were obvious among the subjects with Chlamydia *trachomatis* infection (+) compared to the group without chlamydial infection; elevated level of phyla *Bacteroidetes* was detected in the *Chlamydia trachomatis*-infected samples (+) in infertile group	88% of the subjects from the infertile group was infected by *Chlamydia trachomatis*, as opposed to only 28% in the fertile group	No significant correlation between chlamydial infection was detected with demographics such as age, marital status, as well as ethnicity A lower prevalence of genus Megasphaera was detected among the subjects with *Chlamyidia trachomatis* infection (+) in comparison to the non-infected group

## Data Availability

The data presented in this study are available on request from the corresponding author.

## References

[1] Davenport E.R., Sanders J.G., Song S.J., Amato K.R., Clark A.G., Knight R. (2017). The human microbiome in evolution. BMC Biol..

[2] Knight R., Callewaert C., Marotz C., Hyde E.R., Debelius J., McDonald D., Sogin M.L. (2017). The Microbiome and Human Biology. Annu. Rev. Genom. Hum. Genet..

[3] Maruvada P., Leone V., Kaplan L.M., Chang E.B. (2017). The human microbiome and obesity: Moving beyond associations. Cell Host Microbe.

[4] Whipps J.M., Lewis K., Cooke R.C., Burge N.M. (1988). Mycoparasitism and plant disease control 161–187. Fungi in Biological Control Systems.

[5] Prescott S.L. (2017). History of medicine: Origin of the term microbiome and why it matters. Hum. Microbiome J..

[6] Valenti P., Rosa L., Capobianco D., Lepanto M.S., Schiavi E., Cutone A., Paesano R., Mastromarino P. (2018). Role of lactobacilli and lactoferrin in the mucosal cervicovaginal defense. Front. Immunol..

[7] Moreno I., Simon C. (2018). Relevance of assessing the uterine microbiota in infertility. Fertil. Steril..

[8] Tagini F., Greub G. (2017). Bacterial genome sequencing in clinical microbiology: A pathogen-oriented review. Eur. J. Clin. Microbiol. Infect. Dis..

[9] Proctor L.M., Creasy H.H., Fettweis J.M. (2019). The integrative human microbiome project. Nature.

[10] Buchta V. (2018). Vaginal microbiome. Ces. Gynekol..

[11] Moher D., Liberati A., Tetzlaff J., Altman D.G. (2009). Preferred reporting items for systematic reviews and meta-analyses: The PRISMA statement. BMJ.

[12] Hok T. (1967). Comparative endocervical bacteriology of the mucus. Am. J. Obstet. Gynecol..

[13] Moberg P., Eneroth P., Harlin J., Ljung-wadstrm A., Nord C. (1978). Cervical bacterial flora in infertile and pregnant women. Med. Microbiol. Immunol..

[14] Koskimies A., Paavonen J., Meyer B., Kajanoja P. (1981). Cervicitis and infertility. Am. J. Reprod. Immunol..

[15] Taylor P., Ilesanmi O.A., Edozien L.C. (1995). Culture of the endometrium of infertile women culture of the endometrium of infertile women. J. Obstet. Gynaecol..

[16] Fanchin R., Harmas A., Benaoudia F., Lundkvist U., Olivennes F., Frydman R. (1998). Microbial flora of the cervix assessed at the time of embryo transfer adversely affects in vitro fertilization outcome. Fertil. Steril..

[17] Salim R., Ben-Shlomo I., Colodner R., Keness Y., Shalev E. (2002). Bacterial colonization of the uterine cervix and success rate in assisted reproduction: Results of a prospective survey. Hum. Reprod..

[18] Borovkova N., Korrovits P., Ausmees K., Türk S., Jõers K., Punab M., Mändar R. (2011). Anaerobe in fluence of sexual intercourse on genital tract microbiota in infertile couples. Anaerobe.

[19] Mangot-Bertrand J., Fenollar F., Bretelle F., Gamerre M., Raoult D., Courbiere B. (2012). Molecular diagnosis of bacterial vaginosis: Impact on IVF outcome. Eur. J. Clin. Microbiol. Infect. Dis..

[20] Pelzer E.S., Allan J.A., Waterhouse M.A., Ross T., Beagley K.W., Knox C.L. (2013). Microorganisms within human follicular fluid: Effects on IVF. PLoS ONE.

[21] Campisciano G., Florian F., D’Eustacchio A., Stanković D., Ricci G., De Seta F., Comar M. (2017). Subclinical alteration of the cervical-vaginal microbiome in women with idiopathic infertility. J. Cell. Physiol..

[22] Verstraelen H., Vilchez-Vargas R., Desimpel F., Jauregui R., Vankeirsbilck N., Weyers S., Verhelst R., De Sutter P., Pieper D.H., Van De Wiele T. (2016). Characterisation of the human uterine microbiome in non-pregnant women through deep sequencing of the V1-2 region of the 16S rRNA gene. PeerJ.

[23] Moreno I., Codoñer F.M., Vilella F., Valbuena D., Martinez-Blanch J.F., Jimenez-Almazán J., Alonso R., Alamá P., Remohí J., Pellicer A. (2016). Evidence that the endometrial microbiota has an effect on implantation success or failure. Am. J. Obstet. Gynecol..

[24] Haahr T., Jensen J., Thomsen L., Duus L., Rygaard K., Humaidan P. (2016). Abnormal vaginal microbiota may be associated with poor reproductive outcomes: A prospective study in IVF patients. Hum. Reprod..

[25] Franasiak J.M., Werner M.D., Juneau C.R., Tao X., Landis J., Zhan Y., Treff N.R., Scott R.T. (2016). Endometrial microbiome at the time of embryo transfer: Next-generation sequencing of the 16S ribosomal subunit. J. Assist. Reprod. Genet..

[26] Sahu M.C., Mishra S.P., Panda R., Patnaik T. (2017). Surveillance of microbial flora for infertility couples in an indian tertiary care teaching hospital. Asian J. Pharm. Clin. Res..

[27] Graspeuntner S., Bohlmann M.K., Gillmann K., Speer R., Kuenzel S., Mark H., Hoellen F., Lettau R., Griesinger G., König I. (2018). Microbiota-based analysis reveals specific bacterial traits and a novel strategy for the diagnosis of infectious infertility. PLoS ONE.

[28] Babu G., Singaravelu B., Srikumar R., Reddy S.V. (2017). Comparative study on the vaginal flora and incidence of asymptomatic vaginosis among healthy women and in women with infertility problems of reproductive age. J. Clin. Diagn. Res..

[29] Tao X., Franasiak J.M., Zhan Y., Scott R.T., Rajchel J., Bedard J., Newby R., Treff N.R., Chu T. (2017). Characterizing the endometrial microbiome by analyzing the ultra-low bacteria from embryo transfer catheter tips in IVF cycles: Next generation sequencing (NGS) analysis of the 16S ribosomal gene. Hum. Microbiome J..

[30] Kyono K., Hashimoto T., Nagai Y., Sakuraba Y. (2018). Analysis of endometrial microbiota by 16S ribosomal RNA gene sequencing among infertile patients: A single-center pilot study. Reprod. Med. Biol..

[31] Wee B.A., Thomas M., Sweeney E.L., Frentiu F.D., Samios M., Ravel J., Gajer P., Myers G., Timms P., Allan J.A. (2018). A retrospective pilot study to determine whether the reproductive tract microbiota differs between women with a history of infertility and fertile women. Aust. NZ J. Obstet. Gynaecol..

[32] Koedooder R., Singer M., Schoenmakers S. (2019). The vaginal microbiome as a predictor for outcome of in vitro fertilization with or without intracytoplasmic sperm injection: A prospective study. Hum. Reprod..

[33] Bernabeu A., Lledo B., Díaz M.C., Lozano F.M., Ruiz V., Fuentes A., Lopez-Pineda A., Moliner B., Castillo J.C., Ortiz J.A. (2019). Effect of the vaginal microbiome on the pregnancy rate in women receiving assisted reproductive treatment. J. Assist. Reprod. Genet..

[34] Liu Y., Ko E.Y.-L., Wong K.K.-W., Chen X., Cheung W.-C., Law T.S.-M., Chung J., Tsui S.K.-W., Li T.C., Chim S.S.-C. (2019). Endometrial microbiota in infertile women with and without chronic endometritis as diagnosed using a quantitative and reference range-based method. Fertil. Steril..

[35] Kitaya K., Nagai Y., Arai W., Sakuraba Y., Ishikawa T. (2019). Characterization of Microbiota in Endometrial Fluid and Vaginal Secretions in Infertile Women with Repeated Implantation Failure. Mediat. Inflamm..

[36] Cheong H.C., Yap P.S.X., Chong C.W., Cheok Y.Y., Lee C.Y.Q., Tan G.M.Y., Sulaiman S., Hassan J., Sabet N.S., Looi C.Y. (2019). Diversity of endocervical microbiota associated with genital Chlamydia trachomatis infection and infertility among women visiting obstetrics and gynecology clinics in Malaysia. PLoS ONE.

[37] Lynch T., Peirano G., Lloyd T., Read R., Carter J., Chu A., Shaman J.A., Jarvis J.P., Diamond E., Ijaz U.Z. (2019). Molecular diagnosis of vaginitis: Comparing quantitative pcr and microbiome profiling approaches to current microscopy scoring. J. Clin. Microbiol..

[38] Yarza P., Yilmaz P., Pruesse E., Glöckner F.O., Ludwig W., Schleifer K.-H., Whitman W., Euzéby J., Amann R., Rossello-Mora R. (2014). Uniting the classification of cultured and uncultured bacteria and archaea using 16S rRNA gene sequences. Nat. Rev. Microbiol..

[39] Kralik P., Ricchi M. (2017). A basic guide to real time pcr in microbial diagnostics: Definitions, parameters, and everything. Front. Microbiol..

[40] Weinstock G.M. (2012). Genomic approaches to studying the human microbiota. Nature.

[41] Budding A.E., Grasman M.E., Lin F. (2010). IS-pro: High-throughput molecular fingerprinting of the intestinal microbiota. FASEB J..

[42] Lum D., Guido R., Rodriguez E., Lee T., Mansuria S., D’Ambrosio L., Austin R.M. (2014). Brush Cytology of the Fallopian Tube and Implications in Ovarian Cancer Screening. J. Minim. Invasive Gynecol..

[43] Török P., Molnár S., Herman T., Jashanjeet S., Lampé R., Riemma G., Vitale S.G. (2020). Fallopian tubal obstruction is associated with increased pain experienced during office hysteroscopy: A retrospective study. Updat. Surg..

[44] Messalli E.M., Grauso F., Balbi G., Napolitano A., Seguino E., Torella M. (2013). Borderline ovarian tumors: Features and controversial aspects. Eur. J. Obstet. Gynecol. Reprod. Biol..

[45] Gizzo S., Noventa M., Quaranta M., Vitagliano A., Saccardi C., Litta P., Antona D. (2017). A Novel Hysteroscopic Approach for Ovarian Cancer Screening/Early Diagnosis [Internet] Oncology Letters.

[46] Giampaolino P., Foreste V., Di Filippo C., Gallo A., Mercorio A., Serafino P., Improda F., Verrazzo P., Zara G., Buonfantino C. (2021). Microbiome and PCOS: State-Of-Art and Future Aspects. Int. J. Mol. Sci..

[47] Chiofalo B., Palmara V., Vilos G.A., Pacheco L.A., Lasmar R.B., Shawki O., Giacobbe V., Alibrandi A., Di Guardo F., Vitale S.G. (2021). Reproductive outcomes of infertile women undergoing “see and treat” office hysteroscopy: A retrospective observational study. Minim. Invasive Ther. Allied Technol..

[48] Vitale S.G., Haimovich S., Riemma G., Ludwin A., Zizolfi B., De Angelis M.C., Carugno J. (2021). Innovations in hysteroscopic surgery: Expanding the meaning of “in-office". Minim. Invasive Ther. Allied Technol..

[49] Vitale S.G. (2020). The Biopsy Snake Grasper Sec. VITALE: A New Tool for Office Hysteroscopy. J. Minim. Invasive Gynecol..

[50] Shi Y., Zhu Y., Fan S., Vitagliano A., Liu X., Liao Y., Liang Y., Vitale S.G. (2020). Clinical Characteristics and Antifungal Susceptibility of Candida nivariensis from Vulvovaginal Candidiasis. Gynecol. Obstet. Investig..

